# Head-Mounted Display-Assisted Spatial Guidance in Ultrasonography: A Technical Report

**DOI:** 10.7759/cureus.91792

**Published:** 2025-09-07

**Authors:** Kento Odaka, Kotaro Tachizawa, Masahide Koyachi, Keisuke Sugahara, Satoru Matsunaga, Maki Sugimoto, Akira Katakura

**Affiliations:** 1 Oral and Maxillofacial Radiology, Tokyo Dental College, Tokyo, JPN; 2 Oral Pathobiological Science and Surgery, Tokyo Dental College, Tokyo, JPN; 3 Anatomy, Tokyo Dental College, Tokyo, JPN; 4 Innovation Lab, Teikyo University Okinaga Research Institute, Tokyo, JPN

**Keywords:** head-mounted display, mixed reality in medicine, phantom model, ultrasonography (us), ultrasound probe training

## Abstract

Ultrasonography (US) is a widely used, real-time imaging modality; however, its effectiveness in the head and neck region can be limited by the complexity of anatomical structures and the operator's experience. Mixed reality (MR) technology, utilizing head-mounted displays (HMDs) to overlay 3D holographic models onto the real environment, has shown potential in medical applications. This preliminary study aims to evaluate a prototype HMD-based system that supports US examinations by providing enhanced spatial guidance through MR visualization.

A soft tissue phantom embedded with artificial lesions was scanned with CT to create DICOM datasets, which were reconstructed into 3D holographic models. These models were displayed via a custom MR application developed in Unity Technologies (San Francisco, CA, USA) and operated on Microsoft HoloLens 2 (Microsoft Corp., Redmond, WA, USA). Six sonographers were assigned to two groups: Group A (conventional US) and Group B (HMD-assisted US). Each participant performed a scanning task to identify target lesions. Task performance was assessed using two quantitative metrics: the time to complete the scanning task and the number of missed or falsely identified lesions. Statistical analysis was performed using a t-test, with significance set at p < 0.05.

Group A relied more on wide scanning and screen observation, whereas Group B focused on holographically indicated regions. The mean task times were 178 seconds for Group A and 293 seconds for Group B, with no significant difference (p = 0.21). Clinical experience was negatively correlated with task time (R = -0.71), indicating that more experienced operators completed the task more quickly. Although Group B showed a trend toward fewer missed or false-positive lesions, this did not reach statistical significance (p = 0.076).

The HMD-based MR guidance system provides a feasible approach to support lesion targeting in US examinations. The findings from this preliminary phantom study suggest that MR technology may complement conventional US by enhancing spatial awareness during scanning procedures.

## Introduction

Ultrasonography (US) is a widely used imaging modality for visualizing soft tissue lesions due to its real-time imaging capability and relatively low cost [1,2]. Despite advances in transducer design, the shape and sensitivity of the probe used to generate and receive sound waves, resolution, and portability, US examination of the head and neck remains particularly challenging because of the complexity of anatomical structures and the reliance on operator expertise [3]. To achieve accurate imaging and diagnosis, improving the technical skills and spatial understanding of sonographers is critical.

While US is also employed in therapeutic applications, for instance, to promote tissue healing or drug delivery through focused or pulsed waves [4], this study focuses specifically on its diagnostic role and the challenges of spatial interpretation in real-time imaging.

Various discussions are going on regarding the skillset required to improve US examination. Nicholls et al. emphasized the importance of psychomotor skills in US, including the ability to interpret 3D anatomical structures from 2D images, move the transducer in multiple planes, scan in orthogonal views, and generate optimal images for clinical scenarios [5]. Mulder et al. further noted that visuospatial ability, the skill to perceive shape, position, and movement of anatomical structures, is crucial, mainly since US provides only 2D slices [6]. Operators must mentally reconstruct 3D relationships in real time, which becomes difficult in regions like the neck, where small lymph nodes, vessels, and glands are densely packed. In such complex contexts, spatial misinterpretation and oversight are common, especially among less experienced sonographers.

Various strategies have attempted to improve spatial understanding, particularly in anatomically complex regions such as the head and neck. Landmark-based guidance is useful but experience-dependent. Fusion imaging and electromagnetic tracking offer multi-modal integration but require complex setups and equipment, limiting routine use [7]. Surgical navigation systems help localize lesions but are optimized for fixed scopes, not freehand scanning [8]. These limitations highlight the need for more intuitive and interactive visualization tools to support real-time spatial awareness during sonographic procedures.

In recent years, extended reality (XR) technologies, including virtual reality (VR), augmented reality (AR), and mixed reality (MR), have gained attention for enhancing spatial understanding in medical applications [9]. VR offers immersive training [10,11], while AR overlays virtual content onto real-world scenes. MR combines both, allowing interaction with spatially anchored 3D holograms via head-mounted displays (HMDs). Although most previous studies have applied XR technologies in the context of surgical navigation, such as in orthognathic surgery [12,13], where precise visualization of jaw anatomy is required for surgical planning and navigation, the potential for MR to support US procedures remains underexplored.

Compared to VR-based simulators used for US-guided needle biopsy [14] and vascular access [15], MR offers real-time spatial anchoring that may enhance anatomical orientation during live scanning, rather than only in training. In this study, CT was selected as the imaging source due to its superior spatial resolution for creating detailed holographic models of phantom structures.

This technical report introduces a prototype HMD-assisted US system that overlays CT-derived holographic lesion models onto a phantom’s surface. We assessed feasibility using two quantitative outcomes: (1) task completion time and (2) the number of missed or falsely identified lesions. These findings are intended to provide preliminary insight into the utility of MR integration in sonographic procedures, particularly for improving spatial understanding in real-time settings.

## Technical report

Materials and methods

A phantom model designed for soft tissue biopsy training (Multiple Lesions Phantom, Blue Phantom BPTM130, CAE Healthcare, Mainz, Germany) was used as the specimen. The phantom was constructed from blue, opaque material, ensuring that the internal lesions were not visible through direct visual inspection during scanning. This was confirmed in a pre-task inspection by all participants.

**Figure 1:**
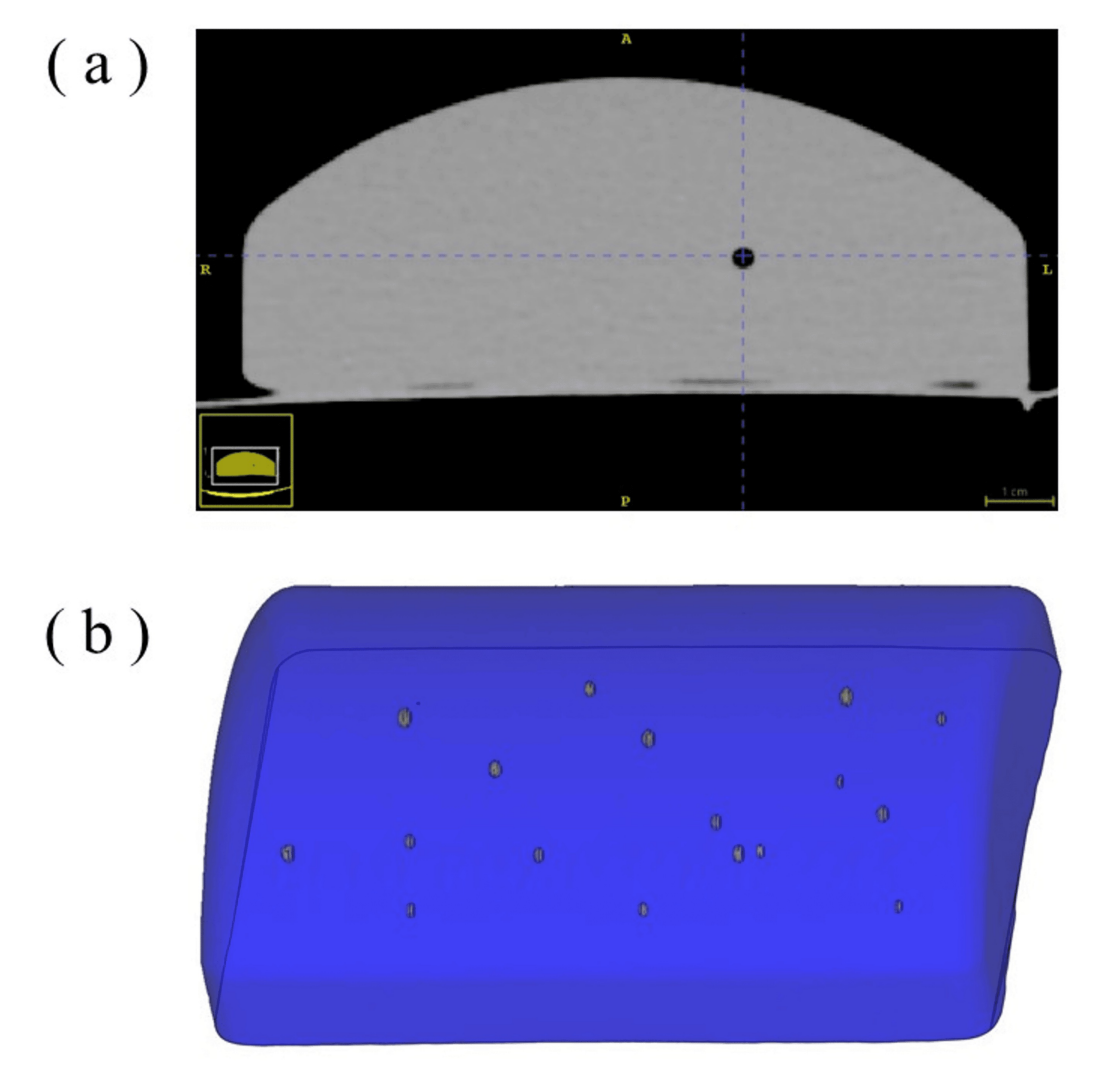
CT-based visualization of the phantom model (a) Axial CT slice showing the internal mass structures. (b) 3D reconstructed model illustrating the spatial arrangement of the masses. CT: computed tomography, 3D: three dimensional

To address the challenge of limited spatial understanding in conventional US, we employed a stepwise technical workflow that included CT scanning, segmentation of anatomical targets, and the construction of a holographic overlay via a custom MR application. Each of these steps was designed to support sonographers in intuitively recognizing the 3D relationships of anatomical structures, thereby reducing reliance on mental reconstruction from 2D images. CT imaging was performed using a Somatom Definition AS scanner (Siemens, Munich, Germany). The acquired images were saved as DICOM data with a voxel resolution of 0.6 mm. During scanning, the phantom was placed inside a plastic container to maintain consistent positioning and shape. Figure 1a shows an axial CT slice depicting the internal mass structures arranged within a confined space. A total of 17 round-shaped mass structures were identified.

The DICOM data were imported into segmentation software (Mimics, Materialise, Leuven, Belgium), where the low-density mass structures were easily segmented. 3D surface models of the phantom and the lesions were generated and exported as STL files. Figure 1b presents a 3D reconstruction created using CAD software (Magics, Materialise, Leuven, Belgium).

An MR application was developed using Unity 2017.4.17 (Unity Technologies, San Francisco, CA, USA), Visual Studio Community 2017 version 15.9.4 (Microsoft Corp., Redmond, WA, USA), and OpenCV 3.1 with the ArUco library (AVA Group, University of Córdoba, Córdoba, Spain). The application visualized the phantom, mass lesions, and a reference marker as a hologram and was deployed on an HMD (HoloLens 2, Microsoft Corp., Redmond, WA, USA).

**Figure 2:**
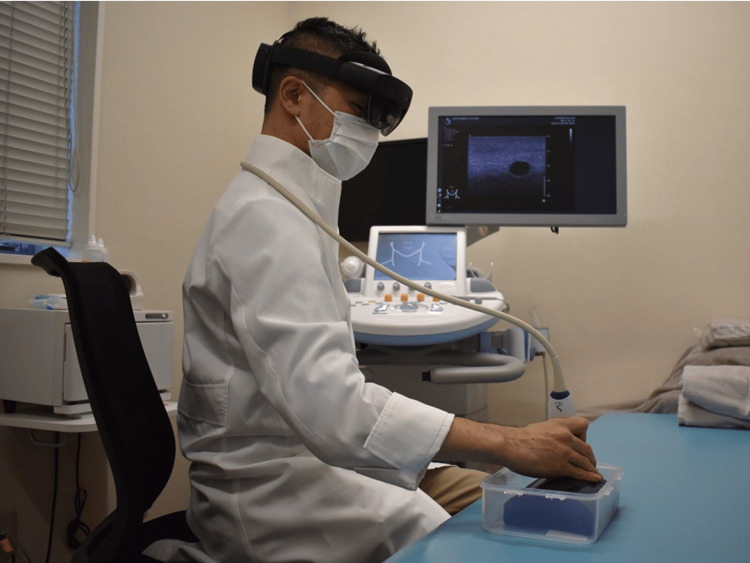
Experimental setup showing the relative positions of the sonographer, phantom, and US machine US: ultrasonography

US images were obtained using a clinical US system (Aixplorer, Konica Minolta, Tokyo, Japan) and were displayed on the system’s built-in monitor, consistent with standard clinical practice. Operators viewed the US images solely on this monitor during the examination. Figure 2 illustrates the setup, including the relative positions of the sonographer, phantom, and US machine. The HMD was used solely to project the holographic representation of the phantom and lesions onto the physical model. To qualitatively evaluate tolerability, participant feedback and behavioral observations were noted during the scanning session (e.g., uninterrupted scanning, absence of discomfort, and natural handling of the probe). These observations were not part of the predefined performance endpoints but were included to assess the practical feasibility of HMD use in a near-clinical setting.

Six sonographers participated in the study and were divided into two groups: Group A (without MR assistance) and Group B (with MR assistance). Participants were divided into two groups (Group A: conventional US, Group B: HMD-assisted US) based on scheduling availability. Randomization was not performed, which resulted in a difference in clinical experience between groups. Both groups wore the HMD, but only Group B used the MR application to visualize lesion locations superimposed on the phantom.

For MR guidance, the 3D CT-based hologram was manually registered to the phantom surface prior to the task. Registration was performed using Holoeyes MD software (Holoeyes, Japan) by aligning the hologram’s position, rotation, and scale to match the external contour of the phantom, referencing the four corners of the phantom viewed vertically. Visual confirmation of registration was performed through the HMD before starting the task. No automatic or marker-based registration system was used, and quantitative measurement of registration error was not conducted. Based on preliminary trials, the positional deviation between the hologram and the phantom surface was estimated to be within approximately 3 mm.

Each sonographer performed a US examination to identify and capture as many mass structures as possible. The task time from initial probe contact to the capture of the final image was recorded using the video recording function of the HMD. An evaluator subsequently reviewed all captured images. Performance was evaluated based on (1) the time required to detect the lesions and (2) the number of missed or falsely identified structures. A t-test was used to compare results between the two groups, with statistical significance defined as p < 0.05. In addition, other qualitative observations, such as scanning behavior and user interaction, were collected during the procedure based on verbal feedback and behavioral impressions noted by the study coordinators.

Results

The average clinical experience of participants was 12.3 years in Group A and 4.7 years in Group B. Randomization was not performed, which resulted in a difference in clinical experience between groups. This imbalance was primarily due to the presence of one highly experienced sonographer in Group A, which may have introduced a performance bias in favor of that group.

All participants completed the scanning task without interruption or signs of discomfort. No complaints were reported regarding the HMD’s fit, weight, or field of view. Additionally, no interference with transducer handling or operator posture was observed, suggesting that the system was physically well tolerated.

**Figure 3:**
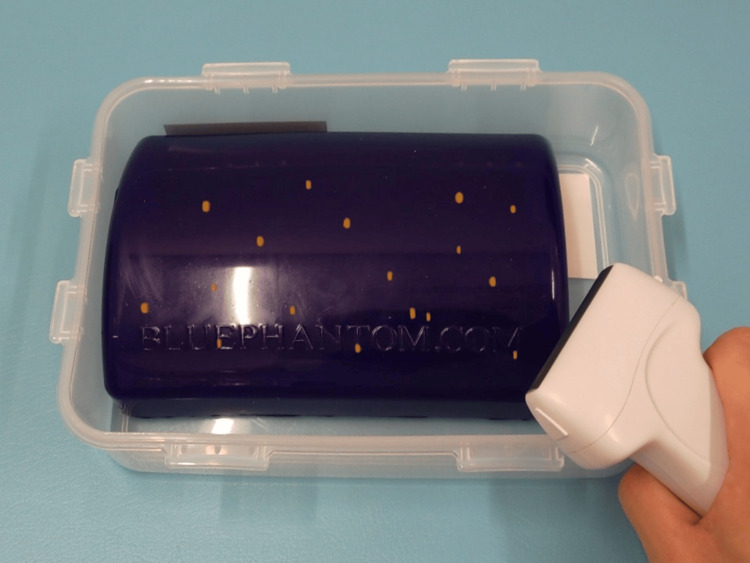
Captured image from the HMD recording function in Group B The mass structures are visualized as yellow holographic objects. HMD: head-mounted display

As shown in Figure 3, video recordings revealed differences in scanning behavior. Group A operators tended to sweep the probe more widely while focusing on the US monitor. In contrast, Group B participants visually located the holographic targets and used a narrower scanning range, guided by HMD projection.

**Figure 4:**
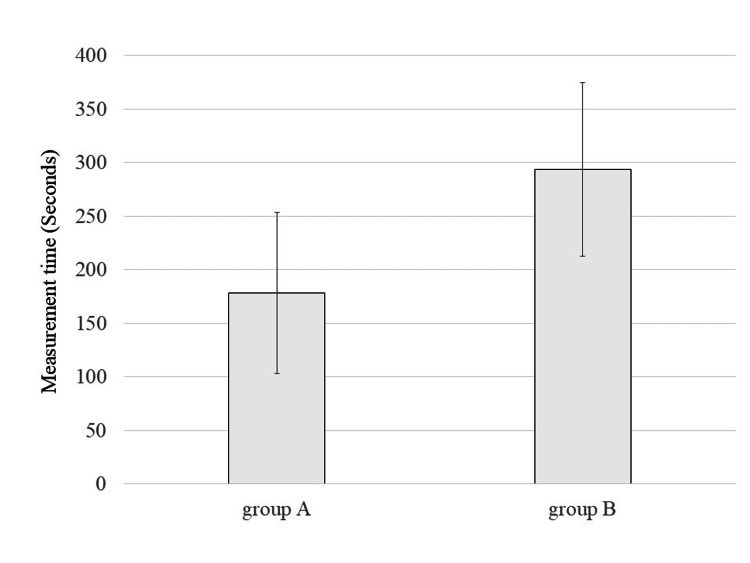
Task completion times for Groups A and B Comparison of mean task completion times between Group A (conventional US) and Group B (HMD-assisted US). Although Group B took longer on average, the difference was not statistically significant (p = 0.21). Error bars indicate standard deviation. US: ultrasonography, HMD: head-mounted display

**Figure 5:**
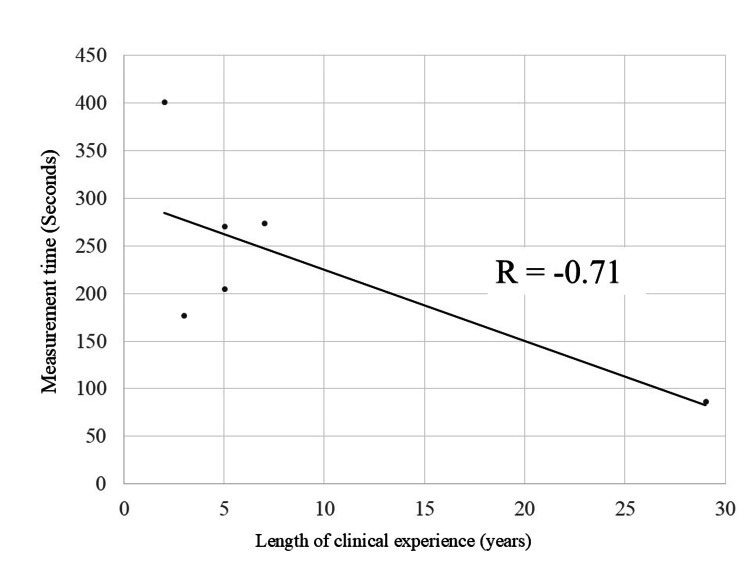
Relationship between task completion time and clinical experience Scatterplot showing a negative correlation between clinical experience (years) and task completion time. Pearson’s r = -0.71.

Figure 4 shows the task completion times for each group. The mean (SD) time was 178.33 (75.12) seconds for Group A and 293.33 (81.17) seconds for Group B. Although no statistical significance was found (p = 0.21), a negative correlation was observed between clinical experience and task time (r = -0.71, Figure 5).

**Figure 6:**
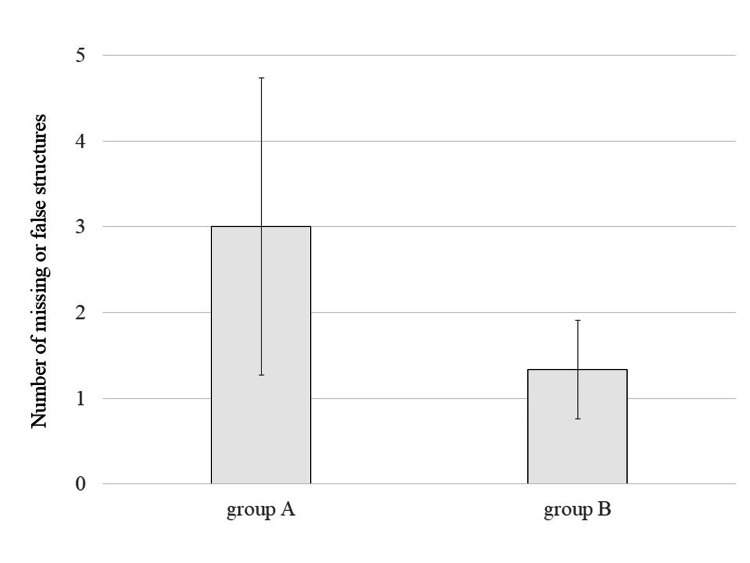
Number of missed or falsely identified structures in Groups A and B Comparison of the number of missed or falsely identified targets between Group A and Group B. Group B exhibited fewer errors on average, though not statistically significant (p = 0.076). Error bars indicate standard deviation.

**Figure 7:**
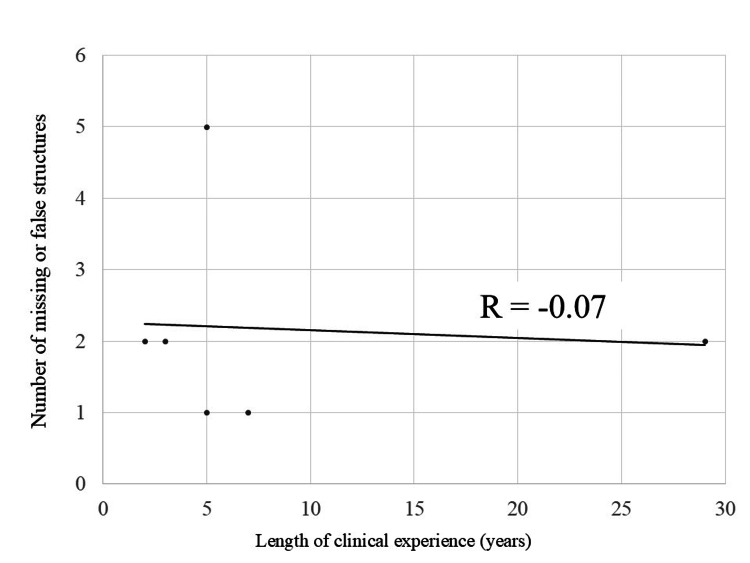
Relationship between the number of missed or false structures and clinical experience Scatterplot illustrating the lack of correlation between clinical experience and number of missed/falsely identified targets. Pearson’s r = -0.07.

Figure 6 presents the number of missed or falsely identified structures. Group B exhibited fewer errors on average, 3.00 (1.73) for Group A vs. 1.33 (0.57) for Group B, but the difference was not statistically significant (p = 0.076). No clear correlation was observed between experience and error rate (r = -0.07, Figure 7).

## Discussion

In the head and neck region, US is commonly used to evaluate various pathologies, including cysts, lipomas, carotid body tumors, hyperplastic lymph nodes, salivary gland tumors, salivary stones, and carcinomas of the tongue or oral cavity [3]. Among these, lymph node assessment is particularly important for cancer control, as lymphatic drainage patterns vary depending on the anatomical subsite within the upper aerodigestive tract [16]. Furthermore, the inclusion of extranodal extension in the American Joint Committee on Cancer Cancer Staging Manual, 8th edition, has emphasized the need for precise morphological information of lymph nodes for treatment planning and management [17]. Consequently, a detailed US evaluation of cervical lymph nodes is routinely performed in the pre- and postoperative settings.

US enables the assessment of lymph node parameters, such as shape, margins, internal structure, and vascularization, which are important in identifying neoplastic involvement [18,19]. While preoperative CT or MRI provides useful reference data for lymph node localization, memorizing 2D images before US examinations can lead to oversight. Conversely, manipulating CT or MRI images in parallel during scanning can increase examination time and impose additional mental effort on the operator. In this context, the method introduced in this study, projecting lesion locations onto the patient's surface using an HMD, may reduce the likelihood of overlooking lymph nodes by providing intuitive spatial guidance.

US is inherently a real-time imaging modality, and reducing examination time can alleviate stress on both the patient and the sonographer. Although the present study did not demonstrate a notable difference in task completion time between groups, the results suggest that examination efficiency remains closely linked to clinical experience rather than to HMD assistance alone. Interestingly, fewer missed structures were observed in the group using HMD guidance. This finding implies that visualizing the target structures through the HMD may help maintain operator focus, potentially reducing oversight in complex anatomical regions. Moreover, the number of detection errors did not show a clear association with years of clinical experience in this study, suggesting that lesion detection accuracy may not be solely dependent on operator experience.

Several limitations must be acknowledged. The small number of participating sonographers (n = 6) reflects the exploratory nature of this study and was determined pragmatically rather than through formal sample size calculation. While the observed trends provide preliminary insights, the limited sample size may restrict the generalizability of the findings. Additionally, participants were allocated into groups based on availability rather than randomization, resulting in unequal clinical experience between groups (12.3 years vs. 4.7 years on average). One highly experienced operator in Group A disproportionately influenced the group’s average, potentially introducing bias in favor of the non-MR group. Future studies with larger cohorts and formalized study designs will be necessary to validate the utility of HMD-assisted US and to reduce bias through randomization and stratification by clinical experience.

Additionally, the phantom used in this study featured simple, round-shaped superficial targets. In contrast, in clinical practice, cervical lymph nodes are often smaller than 1 cm in diameter, located at variable depths, and oriented unpredictably. Accurately projecting such deep or small structures remains a challenge, given the limitations of current HMD systems in depth perception and the manual registration method employed in this study. Additionally, the phantom did not simulate variations in tissue density or internal heterogeneity, which limited its ability to replicate the complexity of real cervical lymph node pathology. The registration accuracy was not quantitatively validated in this study. The estimated alignment error of approximately 3 mm was based on visual approximation and lacks objective verification, limiting confidence in the spatial precision of the system. Furthermore, because the hologram remains fixed following initial registration, any deformation or displacement of the phantom during scanning is not tracked in real time, potentially reducing spatial accuracy. Future improvements may include automated, depth-aware registration, real-time tracking systems for dynamic hologram alignment, and the use of fiducial markers to enhance spatial accuracy.

Despite these limitations, our findings highlight the potential of HMD-based visualization to support US procedures, particularly for trainees and in anatomically complex areas. By enhancing spatial understanding and providing an intuitive visualization of target structures, this system could reduce the risk of diagnostic oversight.

In addition, future studies could incorporate structured assessments of user perception and preference to better understand the subjective usability and acceptability of MR-guided US systems. This could inform interface design and training strategies for broader clinical integration.

Applying this method in a clinical setting would require segmentation of CT or MRI images to create patient-specific models prior to US examination, which could increase the operator’s workload. Recent developments in deep learning have enabled the automatic annotation of lymph nodes from medical images [20,21], offering potential solutions to streamline this process. However, automated extraction may not perform optimally in cases of atypical lymph node morphology or significant structural changes due to metastasis. Radiologist review would likely remain necessary to ensure diagnostic accuracy.

This system may be particularly valuable in clinical scenarios where CT or MRI imaging is already available, such as oncologic staging, surgical planning, or pre-procedural evaluation. Integrating such imaging into the US workflow could enhance spatial orientation without imposing additional diagnostic burden. Future research should explore broader clinical applications of HMD-assisted US, including real-time image fusion techniques to further enhance usability.

## Conclusions

This technical report presented an HMD-assisted US system using holographic guidance based on CT data. The system was exploratorily evaluated through a phantom study involving six sonographers, comparing MR-assisted and conventional US examinations. Although the MR-guided group exhibited a trend toward fewer missed or falsely identified structures, the difference was not statistically significant. Additionally, less experienced operators tended to benefit more from MR support in terms of spatial focus, which suggests the potential for use in training environments or anatomically complex regions. These findings provide preliminary feasibility insights into the use of MR guidance to enhance spatial recognition and reduce oversight during US examinations. However, several limitations restrict generalizability to clinical practice, including the small sample size, absence of blinding, and the use of simple, superficial, and homogeneous phantom lesions. The system’s technological limitations, such as manual registration and lack of depth tracking, may have also influenced performance outcomes.

MR-assisted US may be particularly valuable in educational settings or in anatomically complex regions where spatial disorientation is common. This study supports its feasibility as a technically implementable adjunct to conventional US. However, further validation is essential. Future research should incorporate real patient data, quantitative validation of registration accuracy, automated or dynamic registration methods, and larger, balanced cohorts to more robustly evaluate its effectiveness, usability, and clinical relevance.
